# Development and validation of a simplified CT volumetry for estimating total liver volume in patients with autosomal dominant polycystic kidney and liver disease

**DOI:** 10.1007/s10157-025-02721-9

**Published:** 2025-07-23

**Authors:** Fumihiko Hattanda, Yusuke Watanabe, Yusuke Sakuhara, Shun Takenaka, Tauro Kawamura, Naoko Matsuoka, Daigo Nakazawa, Yoichi M. Ito, Hiroshi Kondo, Shin Goto, Yoshitaka Isaka, Ken Tsuchiya, Toshio Mochizuki, Satoru Muto, Haruna Kawano, Tatsuya Atsumi, Saori Nishio

**Affiliations:** 1 Department of Rheumatology, Endocrinology and Nephrology, Faculty of Medicine and Graduate School of Medicine, Hokkaido University, Sapporo, Japan; 2 Clinical Research and Medical Innovation Center, Institute of Health Science Innovation for Medical Care, Hokkaido University Hospital, Sapporo, Japan; 3 Department of Diagnostic and Interventional Radiology, Tonan Hospital, Sapporo, Japan; 4 Clinical Research and Medical Innovation Center, Hokkaido University Hospital, Sapporo, Japan; 5 Department of Radiology, Teikyo University School of Medicine, Tokyo, Japan; 6 Division of Clinical Nephrology and Rheumatology, Kidney Research Center, Niigata University Graduate School of Medical and Dental Sciences, Niigata, Japan; 7 Department of Nephrology, Osaka University Graduate School of Medicine, Osaka, Japan; 8 Department of Blood Purification, Tokyo Woman’s Medical University, Tokyo, Japan; 9 Department of Nephrology, Tokyo Woman’s Medical University, Tokyo, Japan; 10 Department of Urology, Juntendo University Nerima Hospital, Tokyo, Japan; 11 Department of Urology, Juntendo University Graduate School of Medicine, Tokyo, Japan; 12 Department of Hemodialysis and Apheresis, Hokkaido University Hospital, North 15, West 7, Kita-ku, Sapporo, Hokkaido 060-8638 Japan

**Keywords:** Polycystic liver disease (PLD), Autosomal dominant polycystic kidney disease (ADPKD), Liver volume, Volumetry

## Abstract

**Background:**

Accurate liver volume measurement is crucial for evaluating liver cyst severity and treatment efficacy in polycystic liver disease (PLD). Previous methods are impractical because they are time-consuming and labor-intensive. This study developed and validated two simplified CT imaging methods: the Bi-axial Simplified Measurement Method (BASiM) and the Quadri-dimensional Simplified Measurement Method (QDSiM).

**Methods:**

This retrospective study analyzed 76 CT images from 26 PLD patients who underwent transarterial hepatic artery embolization (TAE). Images were obtained before TAE, 24 weeks after TAE and during the follow-up period. Liver volumes were measured using semi-automatic volumetry, BASiM, and QDSiM. BASiM calculates liver volume based on cranio-caudal, anterior–posterior, and medial–lateral dimensions, while QDSiM divides the liver into left- and right-side sections. This study assessed inter-assessor reliability, measurement accuracy, volume change rate, and calculation times.

**Results:**

BASiM demonstrated strong inter-assessor reliability (intraclass correlation coefficient [ICC]: 0.991, 95% confidence interval [CI] 0.986–0.994) superior to QDSiM (ICC: 0.851, 95% CI 0.205–0.949). Calibrated liver volumes using BASiM and QDSiM were consistent with semi-automatic volumetry (ICC: 0.924, 95% CI 0.858 to 0.957, and ICC: 0.934, 95% CI 0.806–0.970, respectively). BASiM showed better alignment with volume changes (ICC: 0.835, 95% CI 0.537–0.927) compared to QDSiM (ICC: 0.607, 95% CI 0.203–0.800) and required less measurement time (61 ± 4 s vs. 107 ± 9 s, *p* < 0.01).

**Conclusion:**

BASiM provided superior reliability, accuracy, and efficiency for liver volume measurement in PLD, thus useful for the clinical management of PLD.

**Supplementary Information:**

The online version contains supplementary material available at 10.1007/s10157-025-02721-9.

## Introduction

Polycystic liver disease (PLD) is characterized by the presence of 20 or more liver cysts [1], a common manifestation of autosomal dominant polycystic kidney disease (ADPKD), autosomal recessive polycystic kidney disease (ARPKD) and autosomal dominant polycystic liver disease (ADPLD). ADPKD is the most prevalent inherited kidney disease, mainly related to mutations in *PKD1* and *PKD2* genes, and it affects around 1 in 500 to 1,000 individuals [2]. ARPKD is primarily caused by a mutation in the polycystic kidney and hepatic disease 1*(PKHD1*) gene, with a lower prevalence than ADPKD, affecting around 1 in 20,000 to 40,000 individuals [3]. ADPLD is a rare hereditary liver disease caused by pathogenic variants in *PRKCSH* and *SEC63* genes, occurring in 1 in 158,000 people [4]. In PLD patients, progressive liver cyst formation leads to liver enlargement, compressing the stomach and intestines, ultimately causing digestive symptoms, and impairing Activities of Daily Living (ADL).

Treatment strategies for managing enlarged liver cysts include liver cyst aspiration, ethanol sclerosis and transcatheter hepatic artery embolization (TAE) [1, 5]. In addition, surgical interventions, such as liver cyst fenestration, partial liver resection and liver transplantation, have also been performed [6].

Accurate measurement of liver volume is crucial for assessing cyst severity and treatment effectiveness. The current gold standard for liver volume measurement is manual segmentation of cross-sectional imaging [7]. However, this method is seriously time-consuming and requires significant technical effort despite its accuracy. Furthermore, not all medical facilities have access to workstations. Therefore, a simplified and reliable method for measuring liver volume obtained through more available imaging is needed in clinical practice to assess the disease progression of PLD.

To date, various simplified approaches have been proposed for measuring the liver size in various liver diseases [8–11]. Although these techniques are useful for measuring the volume of anatomically intact livers, there are no reports on measuring liver volume in cases of PLD with hepatomegaly and deviation from the typical liver morphology.

Consequently, we have devised two simplified methods for measuring liver volume in PLD patients using non-contrast-enhanced CT scans of axial sections. This study aimed to validate the accuracy of these novel methods compared to semi-automated volumetric measurement techniques and explore their potential for clinical application.

## Materials and methods

In this retrospective study, we used CT images from a previous multicenter clinical trial to assess changes in liver volume among patients with PLD who received TAE. The results of the previous study are currently being prepared for submission. The institutional review board of our hospital and collaborating hospitals approved the previous clinical trials, with investigators adhering to the Declaration of Helsinki and ethical guidelines and regulations for clinical research in Japan. All patients provided written informed consent to analyze their clinical findings, including CT images, at the time of enrollment in the previous study.

### Patient population and imaging acquisition

In previous clinical trials, we conducted involved 26 patients (4 males, 22 females, mean age 53.8 ± 9.7 years) with PLD who received TAE at our hospital and collaborating facilities from 2015 to 2017. Patients’ characteristics at baseline were described in Table 1 (Table 1). Each patient underwent abdominal non-contrast CT (slice thickness 3–5 mm) at three points: before the procedure, 24 weeks after TAE, and during the follow-up period (36 ± 12 months). While 24 patients received CT scans at all three time points, two patients only received scans before the procedure and 24 weeks after. Thus, we analyzed a total of 76 CT images in this study.

**Table 1:** Characteristics of patients with polycystic liver disease

	*n* = 26
Sex, male, *n* (%)	4 (15.4)
Age (years)	53.8 ± 9.7
Height (cm)	160.2 ± 6.6
Body Weight (kg)	58.7 ± 9.9
Polycystic kidney disease, *n* (%)	24 (92.3)
Polycystic liver disease, *n* (%)	2 (7.7)

Data are described as the mean ± standard deviation and number (percentage) of patients

*n* (%), number (percentage)

### Measurement methods of liver volume

**Fig. 1:**
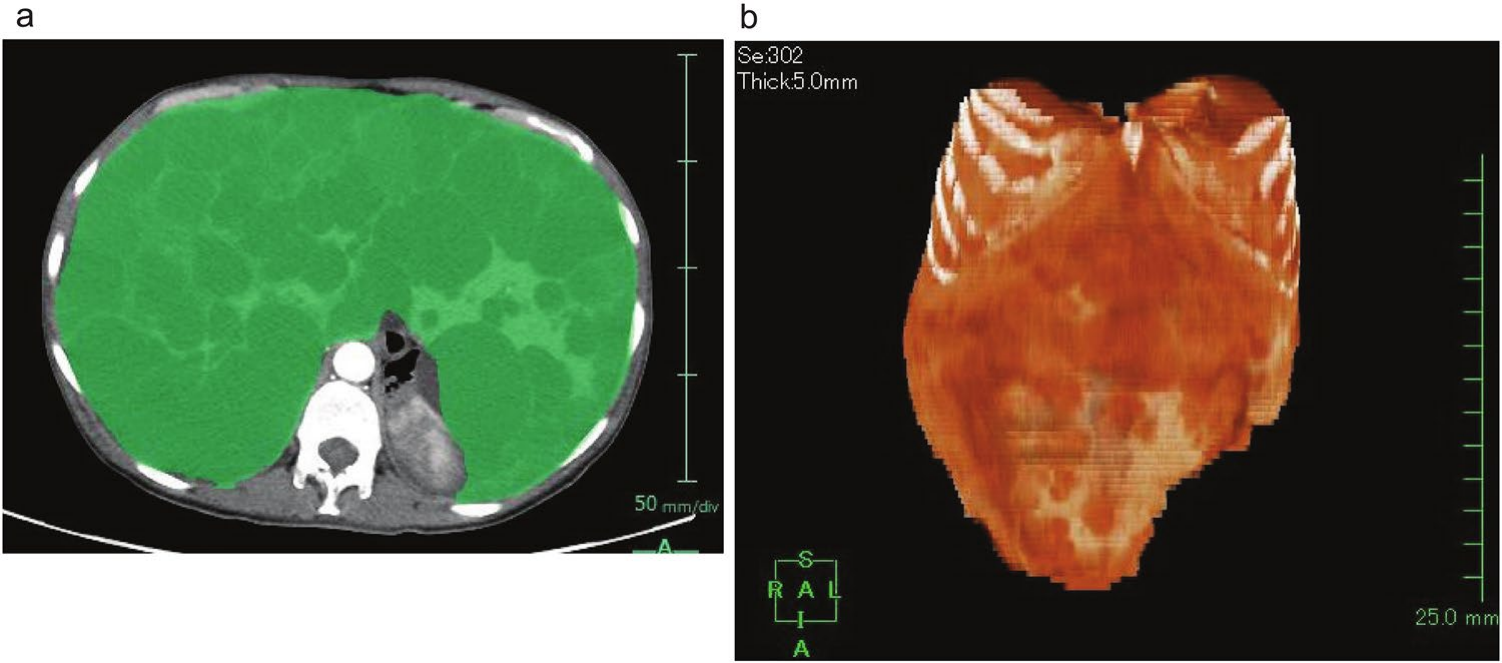
Measurement of liver volume using an image workstation (SYNAPSE VINCENT®, Fujifilm Medical Systems, Tokyo, Japan). The periphery of the liver parenchyma is manually extracted in each CT image slice in the coronal section (3–5 mm thickness) **a** The workstation creates a 3D structure of the whole liver parenchyma, and the liver volume is automatically measured from the extracted area (**b**)

We utilized SYNAPSE VINCENT® software (Fuji Medical Systems, Tokyo, Japan) for semi-automatic liver volume measurement, and these data were used as reference liver volume for this study. This workstation created a 3D structure of the entire liver by manually tracing the periphery of the liver parenchyma on axial CT images. After all the liver slices were plotted, the workstation measured the liver volume automatically (Fig. 1). This measurement method was defined as"semi-automatic volumetry."In the clinical trial, volumetric measurements were conducted in a blinded manner by independent radiologists, and these measurements were utilized as the actual values for further analyses in this study.

After several discussions with a team of internists and radiologists who had substantial experience in managing PLD patients, along with research and development professionals and biostatisticians, we devised two different simplified methods to calculate liver volume index: the Bi-axial Simplified Measurement Method (BASiM) and the Quadri-dimensional simplified Measurement Method (QDSiM). We then estimated the liver volume using both simplified methods based on the same sets of CT data. Each measurement method is described below:

### Bi-axial simplified measurement method (BASiM)

**Fig. 2:**
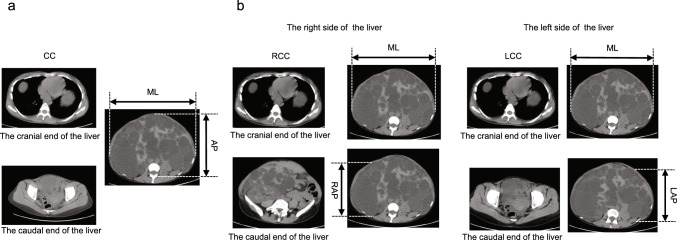
Measurement of liver diameters and liver volume indices using simplified measurement method. **a** Bi-axial Simplified Measurement Method (BASiM): CC is calculated by　multiplying the number of CT image slices by the distance from the head to the foot side. AP is measured by selecting the image slice where the liver protrudes most ventrally. ML is described as the maximum transverse diameter of the left and right abdomen in the same coronal cross-sectional image where the AP is measured. **b** Quadri-dimensional Simplified Measurement Method (QDSiM): LCC and RCC are measured by multiplying the number of image slices by the interval from the head to the foot side on each side. LAP and RAP are calculated by determining the maximum distance from the ventral to the dorsal side. ML is measured by selecting the slice with the longest distance between the left and right margins of the liver. *CC* cranio-caudal distance, *AP* anterior–posterior distance, *ML* medial–lateral distance, *LCC* cranio-caudal distance on the left side, *RCC* cranio-caudal distance on the right side, *LAP* anterior–posterior distance on the left side, *LAP* anterior–posterior distance on the right side

This method calculates the cranio-caudal distance (CC) by counting the axial CT image slices from the cranial to the caudal end of the liver and multiplying by the slice thickness. The anterior–posterior distance (AP) is defined as the distance from the ventral surface of the liver to the junction of the lamina plate and the spinous process of the vertebral body on the slice where the liver protrudes most ventrally. Furthermore, the medial–lateral distance (ML) is determined as the maximum transverse diameter of the liver on the same axial CT slice where the AP was measured (Fig. 2). The BASiM Liver Volume Index was calculated using the formula below:

$${\text{BASiM Liver Volume Index}} = {\text{CC}} \times {\text{AP}} \times {\text{ML}}{.}$$

### Quadri-dimensional simplified measurement method (QDSiM)

This approach divides the liver into left and right sides along the body's median. The cranio-caudal distances for both sides (LCC and RCC) are calculated similarly to BASiM. The anterior–postal distances are measured for each side (LAP and RAP) by determining the maximum distance from the ventral to the dorsal sides. The ML is defined differently from BASiM by selecting the slice with the largest transverse diameter, regardless of the CT slice where LAP and RAP are measured (Fig. 2). The volume indices for each side are calculated as follows:

$$\text{Left Liver Volume Index }\text{=} \, \frac{\text{LCC }\times \text{ LAP }\times \text{ML}}{2}$$

$$\text{Right Liver Volume Index }\text{=} \, \frac{\text{RCC }\times \text{ RAP }\times \text{ML}}{2}$$

$$\text{QDSiM Liver Volume Index }\text{=} \, \text{Left Liver Volume Index}\text{ } + \text{ Right Liver Volume Index.}$$

To assess inter-assessor reliability, a board-certified interventional radiologist and a physician experienced in managing ADPKD and ADPLD independently calculated liver volume indices using BASiM and QDSiM in a blinded manner, without access to the semi-automatic volumetry results or other assessors'data. Additionally, the consistency of the diameter measurements and the liver volume indices obtained from the two assessors were calculated for each method. Furthermore, we analyzed the correlation between the liver volume indices from BASiM and QDSiM with the reference liver volumes obtained from semi-automatic volumetry. Moreover, the liver volume indices were calibrated to match the reference liver volume. This calibration was achieved by applying a formula described in the results section.

It's crucial to assess the liver volume changes based on liver cyst growth or treatment efficacy. The change rate was calculated using the formula below. Furthermore, the consistency of the change rate in liver volume obtained from each measurement method was evaluated.

$${\text{The rate of liver volume change }}\left( \% \right) = \frac{{{\text{Post - treatment liver volume}} - {\text{Pre - treatment liver volume}}}}{{{\text{Pre}}{ - }{\text{treatment liver volume}}}} \times 100.$$

Moreover, this study assessed the time required to measure all distance parameters for calculating the liver volume indices from opening the patient’s image file to completing measuring all parameters.

### Statistical analysis

Data were analyzed using mean ± standard deviation for normally distributed data and median with interquartile range for non-normally distributed data. The liver volume change rate was described as an average value with a 95% confidence interval (95% CI). Spearman's rank correlation coefficients assessed the relationship between reference liver volumes and liver volume indices from BASiM and QDSiM. Intraclass correlation coefficients (ICC), specifically the two-way random-effect single-measure model (ICC (2,1)), evaluated inter-assessor reliability and agreement for measurement values. ICC also assessed the consistency of liver volume and the change rate between semi-automatic volumetry and simplified methods. Agreement levels were further described using Bland–Altman plots [12], which displayed mean differences and 95% limits of agreement as the mean difference of 1.96 SD. Statistical analyses were conducted with SPSS version 28 (IBM Corp., Armonk, NY) in ICC and IMP software version 16.2 (SAS Institute Inc., Cary, NC) in other analyses. *P values* less than 0.05 were considered statistically significant in this study.

## Results

### Inter-assessor reliability of each simplified measurement method

First, we evaluated the degree of agreement between the assessors in the diameters obtained through BASiM and QDSiM. The results showed as follows: for BASiM, the ICCs for diameters CC, AP, and ML were 0.988 (95% CI 0.981–0.992), 0.959 (95% CI 0.903–0.979) and 0.981 (95% CI 0.961–0.989), respectively. In the case of QDSiM, the ICCs for the diameters RCC, LCC, RAP, LAP, and ML were 0.930 (95% CI 0.585–0.975), 0.911 (95% CI 0.851–0.946), 0.862 (95% CI 0.790–0.910), 0.187(95% CI – 0.03 to 0.398), and 0.936 (95% CI 0.880–0.964), respectively (Table 2).

**Table 2:** Diameter and liver volume index were measured by different assessors

	Assessor A	Assessor B	ICC
BASiM			
CC (cm)	30.3 ± 5.2	30.3 ± 5.3	0.988 (0.981–0.992)
AP (cm)	21.4 ± 2.4	21.8 ± 2.4	0.959 (0.903–0.979)
ML (cm)	28.9 ± 2.7	28.6 ± 2.7	0.981 (0.961–0.989)
Liver Volume Index (cm^3^)	18,572.9 (14,037.2–24,442.0)	18,814.0 (14,317.6–24,522.6)	0.991 (0.986–0.994)
QDSiM			
Left side of the liver			
LCC (cm)	21.7 ± 6.4	20.8 ± 6.3	0.911 (0.851–0.946)
LAP (cm)	17.1 ± 4.0	13.8 ± 2.5	0.187 (– 0.039 to 0.398)
Right side of the liver			
RCC (cm)	30.1 ± 5.3	28.7 ± 5.6	0.930 (0.585–0.975)
RAP (cm)	19.0 ± 2.0	18.9 ± 2.1	0.862 (0.790–0.910)
ML (cm)	28.1 ± 2.7	27.7 ± 2.6	0.936 (0.880–0.964)
Liver Volume Index (cm^3^)	13,417.6 (9810.9–16,506.5)	11,700.8 (8202.7–14,901.7)	0.851 (0.205–0.959)

Data are described as mean ± standard deviation, median (interquartile) and intraclass correlation coefficient (95% confidence interval)

*BASiM*, Bi-axial Simplified Measurement Method; *QDSiM*, Quadri-dimensional Simplified Measurement Method, *CC* cranio-caudal distance, *AP* anterior–postal distance, *ML* medial–lateral distance, *LCC* cranio-caudal distance on the left side, *RCC* cranio-caudal distance on the right side, *LAP* anterior–postal distance on the left side, *RAP* anterior–postal distance on the right side, *ICC* intraclass correlation coefficient

### Correlation of reference liver volume and liver volume indices calculated by simplified measurement methods

**Fig. 3:**
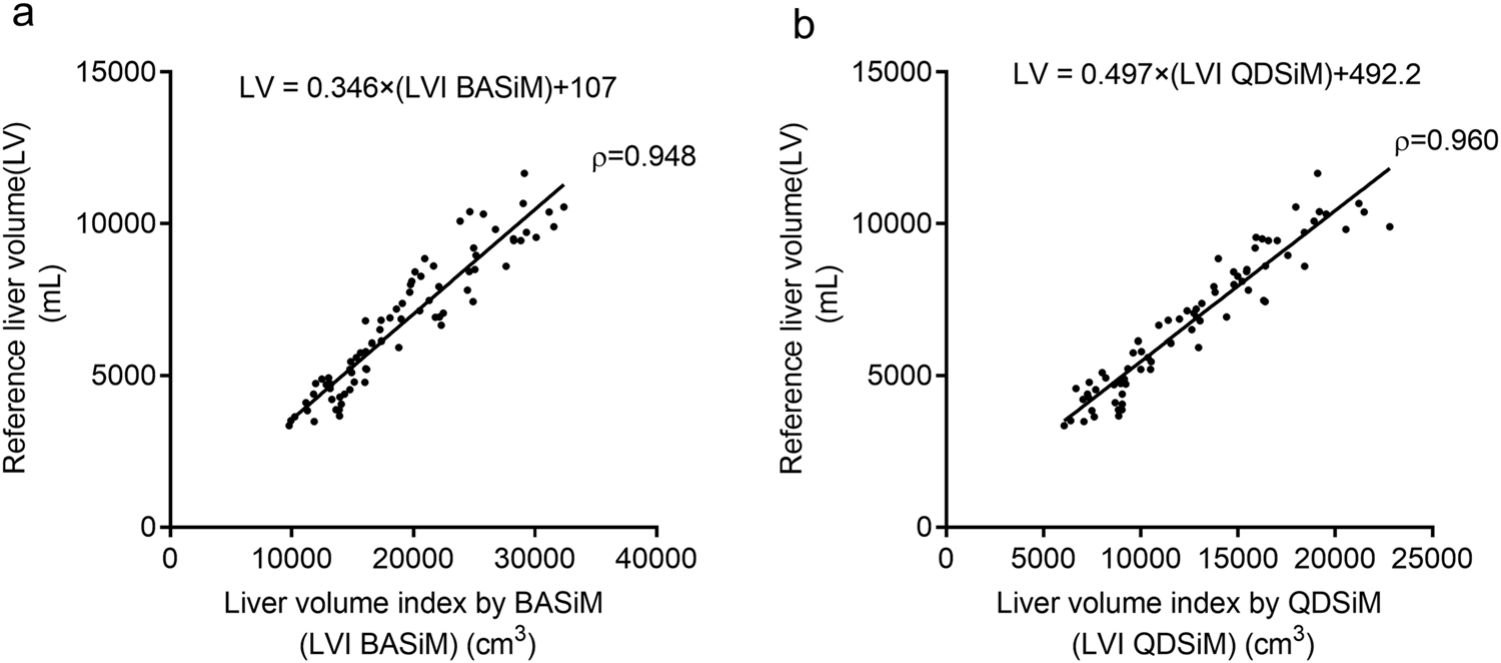
Correlations between liver volume obtained from the semi-automatic volumetry and liver volume indices obtained by two simplified measurement methods. Liver volumes obtained by semi-automatic volumetry strongly correlated with liver volume indices from BASiM (**a**) and QDSiM (**b**). The liver volume indices were calculated as the mean values between assessors A and B. Calibration coefficient was devised based on regression coefficients of liver volume and liver volume index (**a** 0.346, **b** 0.497). Calibration equation was formulated and applied to adjust the volume index to the liver volume

In addition, we assessed the correlation between the reference liver volumes obtained through semi-automatic volumetry and liver volume indices calculated by simplified measurement methods. A significant correlation was revealed between the reference liver volumes and the liver volume indices for BASiM (mean value between assessors A and B) (*ρ* = 0.948, *p* < 0.01) and QDSiM (mean value between assessors A and B) (*ρ* = 0.960, *p* < 0.01) (Fig. 3).

### Calibration of the liver volume index to match the reference liver volume

In our results, the correlation coefficients between reference liver volumes and liver volume indices calculated by BASiM and QDSiM were 0.346 and 0.497, respectively (Fig. 3). Based on these correlation coefficients, the liver volume indices were calibrated by applying the following formula to fit the volume index to the reference liver volume.

$$\text{BASiM Liver Volume}= \frac{\text{BASiM Liver Volume Index}}{3}$$

$$\text{QDSiM Liver Volume}= \frac{\text{QDSiM Liver Volume Index}}{2}$$

The calibrated liver volumes were calculated based on the mean liver volume indices measured by assessors A and B. The reference liver volumes and the calibrated volumes for all images and three time periods: before TAE, 24 weeks after the treatment and during follow-up are presented in Table 3. A detailed summary of all cases, including slice thickness and the distance parameters using BASiM and QDSiM, is described in the supplementary materials (Supplemental Tables 1 and 2).

**Table 3:** Liver volumes using different methods, by different assessors, or during different observation periods

Measurement method		Liver volume (mL)		
	Total	Before TAE	24 weeks after TAE	During the follow-up period
Semi-automated volumetry	6833.8 (4753.6–8565.3)	7047.5 (4865.5–8594.5)	6840.5 (4613.3–8306.0)	6733.2 (4629.2–9107.9)
BASiM				
Assessor A	6191.0 (4679.1–8147.3)	6360.4 (4875.0–8083.9)	5675.3 (4588.4–8046.1)	6119.7 (4740.7–8216.1)
Assessor B	6271.3 (4772.5–8181.4)	6456.5 (4828.7–8010.6)	5757.4 (4711.9–8251.0)	6063.2 (4802.1–8332.2)
Mean value	6237.0 (4711.8–8188.3)	6416.2 (4870.4–8047.2)	5692.8 (4650.7–8205.5)	6062.7 (4819.2–8274.2)
QDSiM				
Assessor A	6708.8 (4905.5–8253.2)	7302.4 (5010.5–8768.9)	6708.8 (4906.5–8568.0)	5654.7 (4087.0–8078.6)
Assessor B	5850.4 (4101.3–7450.9)	5908.0 (4262.2–7971.1)	5856.2 (4085.3–7256.4)	5479.5 (3940.3–7450.9)
Mean value	6340.9 (4497.6–7957.0)	6460.6 (4568.3–8457.6)	6388.9 (4517.5–7840.3)	5586.4 (3889.2–7898.3)

Data are described as the median (interquartile range)

*BASiM* Bi-axial Simplified Measurement Method, *QDSiM* Quadri-dimensional Simplified Measurement Method

### Comparison between reference liver volume and calibrated liver volume obtained through simplified measurement methods

**Fig. 4:**
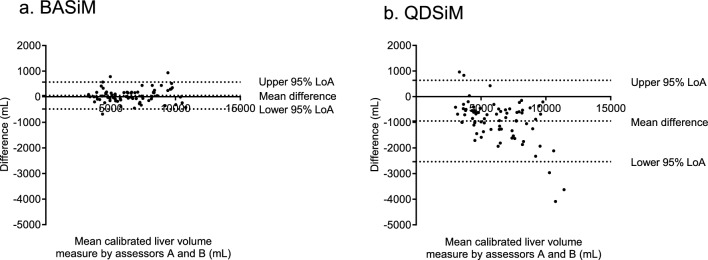
Bland–Altman plots for agreement of calibrated liver volumes between assessors A and B based on BASiM (**a**) and QDSiM (**b**). The plot showed that BASiM was more consistent in measuring liver volumes between the two assessors compared to QDSiM. The 95% limits of agreement (LoA) are defined as the mean difference ± 1.96 standard deviations (SD)

**Fig. 5:**
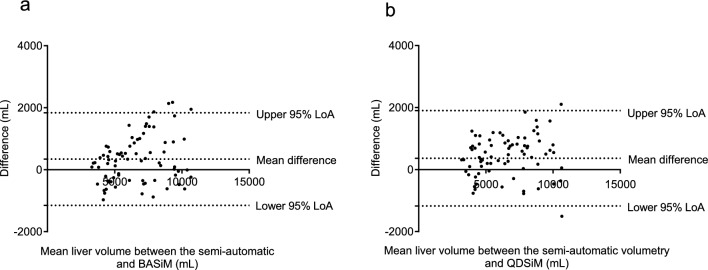
Bland–Altman plots for agreement between liver volumes measured by a semi-automatic volumetry and two simple measurement methods, BASiM (**a**) and QDSiM (**b**). The plots revealed that BASiM and QDSiM were highly consistent with the semi-automatic volumetry in determining liver volume. 95% LoA, 95% limits of agreement as the mean difference of 1.96 SD

To assess the accuracy of calibrated liver volume measurements, the inter-assessor agreement of simple measurement methods between assessors A and B was evaluated. The results showed that the ICCs of liver volume were 0.991 (95% CI 0.986 to 0.994) for BASiM, and 0.851 (95% CI 0.205–0.949) for QDSiM. To compare the differences in liver volume values calculated by the assessors, we created Bland–Altman plots (Fig. 4). These plots showed that the mean difference between the two measurements by assessors A and B was 46.7 mL for BASiM and – 946.6 mL for QDSiM, respectively. On the consistency of liver volume measurements, the ICC between reference liver volume and calibrated liver volumes through BASiM (mean value between the assessors) was 0.924 (95% CI 0.858–0.957), and between reference liver volume and QDSiM (mean value between assessors) was 0.934 (95% CI 0.806–0.970). The Bland–Altman plots further revealed that the mean difference in liver volume values between reference liver volume and BASiM was 341.9 mL, whereas between reference liver volume and QDSiM was 450.7 mL (Fig. 5). Additionally, the Bland–Altman plots at three-time points are shown in the supplementary materials (Supplemental Fig. 1).

### Consistency of liver volume measurements across different CT slice thicknesses

We assessed the consistency of liver volume measurements at different slice thicknesses from the same CT scan. Among the 76 CT images included in this study, 12 contained both 5 mm and 3 mm slices. The agreement between liver volume measurements from 5 and 3 mm slices of the same CT scan was analyzed using the Bland–Altman plots with the results presented in the supplementary materials (Supplemental Fig. 2).

### Comparison of the rate of change in liver volume

**Fig. 6:**
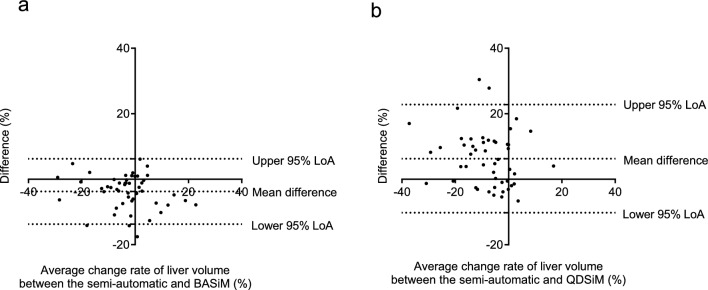
Bland–Altman plots for agreement between change rates of liver volumes measured by a semi-automatic volumetry and two simplified measurement methods, BASiM (**a**) and QDSiM (**b**). The analysis indicated that the change rate based on BASiM was more consistent with the semi-automatic method than QDSiM. 95% LoA, 95% limits of agreement as the mean difference of 1.96 SD

The rate of change in liver volume from baseline to 24 weeks after TAE was – 6.05% (95% CI – 9.25 to – 2.85) using semi-automatic volumetry, – 3.69% (95% CI – 6.78 to – 0.60) using BASiM (mean value between assessors), and – 7.15% (95% CI – 10.6 to – 3.71) using QDSiM (mean value between assessors). In addition, the change rate in liver volume from baseline to the follow-up period was – 3.35% (95% CI – 8.47 to 1.76), 1.89% (95% CI – 3.79 to 7.57), and – 15.27% (95% CI – 20.86 to – 9.67), respectively. Furthermore, the ICC of the change rate in liver volume obtained through semi-automatic volumetry and BASiM was 0.835 (95% CI 0.530 to 0.927), while the ICC of liver volume by semi-automatic volumetry and QDSiM was 0.607 (95% CI 0.203 to 0.800) (Table 4). Moreover, the Bland–Altman plot revealed that the mean difference in the liver volume change rate between semi-automatic volumetry and BASiM and QDSiM was – 3.75 mL and 6.29 mL, respectively (Fig. 6).

### Measurement time for all the distance parameters using the simplified measurement methods

Regarding BASiM, the time taken to measure all distance parameters, including CC, AP, and ML, was 61 ± 6 s for assessor A and 61 ± 6 s for assessor B, respectively. In the case of QDSiM, the time taken to measure all distance parameters, including LCC, LAP, RCC, RAP, and ML, was 109 ± 9 s for assessor A and 106 ± 14 s for assessor B, respectively. The average measurement time taken for liver volume between assessors A and B was 61 ± 4 s in BASiM and 107 ± 9 s in QDSiM. The measurement time was significantly shorter with BASiM than QDSiM (*p* < 0.01).

## Discussion

In this study, we developed two simplified measurement methods, BASiM and QDSiM, to estimate the volume of enlarged livers with multiple cysts in PLD patients. Our results revealed that BASiM demonstrates higher inter-assessor reliability, a stronger consistency with semi-automatic volumetry in measuring liver volume, and a significantly shorter measurement time than QDSiM. Furthermore, there was a strong agreement between BASiM and semi-automatic volumetry regarding the rate of change in liver volume, indicating that BASiM is a plausible alternative measurement for clinical use.

The most accurate method to evaluate liver volume involves manually tracing the margin of the liver on all slice images of the liver using a workstation [7]. This study utilized semi-automatic volumetry as an accurate liver volume. Although semi-automatic volumetry can measure the actual liver volume, this approach typically requires significant time and technical effort. In recent years, since imaging technology has significantly advanced, various reliable methods have been reported for fully automatic liver volume measurement, including deep learning techniques with automatic intelligence [13–16]. These automated measurement workstations can provide an easy-and-quick way to measure liver volume. However, it would be challenging for physicians to measure liver volume using such a sophisticated technology in clinical practice. Additionally, patients with PLD may exhibit a significantly enlarged liver size and distorted liver morphology, making it difficult for fully automatic methods to identify the outer edge of the liver and measure liver volume accurately. In contrast, our simplified measurement methods involve measuring only three or five dimensions of non-enhanced CT images, making them feasible methods for clinical practice.

Previous studies have explored simple methods for estimating liver volumes by measuring the liver's diameter (liver width, thickness, and height) using ultrasound, CT or magnetic resonance imaging (MRI) [10, 17, 18]. Roloff et al. [10] demonstrated a strong linear correlation between liver volume indices from CT images and accurate volumes measured by semi-automatic volumetry. Furthermore, they estimated the calibrated liver volume by dividing the liver volume index by a calibration factor of 3.6 and found that the calibrated liver volume was highly consistent with the accurate liver volume. Although Roloff's measurement technique is convenient for clinical practice, its accuracy is uncertain for polycystic livers due to a significantly larger size of liver and the variations in shape between polycystic and normal livers, which complicates accurate margin definition and diameter measurement. Our study introduces two distinct measurement approaches: BASiM and QDSiM. BASiM involves assessing the three diameters of liver as in the previous report [10]. To ensure reproducibility among the assessors and simplify the process, we selected the same CT slice images for both AP and ML. On the other hand, QDSiM estimates liver volume by dividing liver into left and right sides. This approach considers the inherent imbalance in size between left and right sides, which differs from that of a normal liver.

BASiM demonstrates high inter-assessor agreement for all diameters and calculated liver volumes. Furthermore, the calibrated liver volume obtained from BASiM showed a high consistency with the reference liver volumes. Moreover, liver volume values calculated by BASiM from both 5 mm and 3 mm slices of the same CT scan were highly consistent (Supplemental Fig. 2). These findings suggest that BASiM is a reliable and practical method for assessing enlarged livers with multiple cysts. In contrast, QDSiM showed a lower consistency in diameter measurements compared to BASiM. Although semi-automatic volumetry and QDSiM showed a high accuracy in liver volumes, LAP has a lower inter-assessor reliability in QDSiM, which could lead to decreased accuracy in liver volume values across different assessors. In QDSiM, LAP is defined as the maximum front-to-back distance of the left side of liver, mainly composed of the left lobe. As the left lobe is smaller than the right lobe, the development of multiple cysts in the left lobe can alter its shape, leading to significant deviations from its original formation. Significant deformation of the left lobe due to cysts can cause variations in LAP among different assessors, resulting in a low agreement between measurements. In addition, the number of measurement items required to calculate liver volume is higher than in QDSiM, which may lead to inaccurate measurements.

It is crucial to evaluate the changes in liver volume in patients with PLD to determine the effectiveness of the treatment. This study also compared the liver volume change rates before and during the 24-week treatment or follow-up periods after TAE. The results revealed that BASiM has a solid agreement with the semi-automatic volumetry in determining the change rate in liver volume. In contrast, while the semi-automatic volumetry and the QDSiM demonstrated some degree of consistency, they were not as reliable as BASiM. These findings suggest that BASiM has better accuracy for estimating liver volume and its changes in PLD patients compare to QDSiM.

This study has several limitations. First, the sample size and the number of CT images were relatively small, and variations in liver volume and shape in PLD due to differences in cyst distribution were not fully addressed. Second, BASiM and QDSiM rely solely on CT imaging and have not been tested for accuracy with other imaging modalities, such as ultrasonography or MRI. Finally, this study only assessed liver volumes in advanced PLD patients with enlarged hepatic cysts requiring hepatic TAE. Therefore, it remains unclear whether this simplified CT volumetry method can accurately estimate liver volume in patients with smaller livers or those without hepatic cysts.

In conclusion, BASiM developed in this study adequately estimates the liver volume and the changes in patients with PLD by measuring the liver diameter on CT images. While there are some limitations, this method can be utilized as a practical alternative measurement tool in clinical settings. It definitely helps physicians enhance clinical management and research.

## Supplementary Information

Below is the link to the electronic supplementary material.

Supplemental Fig. 1 Bland–Altman plots for agreement between liver volumes measured by a semi-automatic volumetry and two simple measurement methods: the Bi-axial Simplified Measurement Method (BASiM) (a) and the Quadri-Dimensional Simplified Measurement Method (QDSiM) (b) at three time points: before TAE, 24 weeks after TAE, and during the follow-up period. The mean difference in liver volume between a semi-automatic volumetry and BASiM (mean value between assessors A and B) was 482.3 mL before TAE, 324.1 mL 24 weeks after TAE and 209 mL during the follow-up period, respectively. The mean difference in liver volume between a semi-automatic volumetry and QDSiM (mean value between assessors A and B) was 156.0 mL before TAE, 228.3 mL 24 weeks after TAE and 1011 mL during the follow-up period, respectively. The 95% limits of agreement (LoA) are defined as the mean difference ± 1.96 standard deviations (SD)

Supplemental Fig. 2 Bland–Altman plots showing the agreement of calibrated liver volume measurements between 5 mm and 3 mm slices using BASiM (a) and QDSiM (b). In BASiM, the mean difference in calibrated liver volume between the 5 mm and 3 mm slices measured by Assessor A was – 2.84 mL, and by Assessor B was – 9.74 mL. In contrast, in QDSiM, the mean difference in calibrated liver volume between the 5 mm and 3 mm slices measured by Assessor A was 189.7 mL, and by Assessor B was 295.7 mL. The results show a high consistency in liver volume values calculated by BASiM for both 5 mm and 3 mm slices. In contrast, QDSiM demonstrates a lower consistency compared to BASiM. The 95% LoA are defined as the mean difference ± 1.96 SD

Supplementary file3 (DOCX 58 KB)

## References

[1] Drenth JP, Chrispijn M, Nagorney DM, Kamath PS, Torres VE. Medical and surgical treatment options for polycystic liver disease. Hepatology. 2010;52(6):2223–30. 21105111 10.1002/hep.24036

[2] Torres VE, Harris PC, Pirson Y. Autosomal dominant polycystic kidney disease. Lancet. 2007;369(9569):1287–301. 17434405 10.1016/S0140-6736(07)60601-1

[3] Patil A, Sweeney WE, Jr., Avner ED, Pan C. Childhood Polycystic Kidney Disease. In: Li X, editor. Polycystic Kidney Disease. Brisbane (AU): Codon Publications Copyright: The Authors.; 2015. 27512782

[4] Perugorria MJ, Masyuk TV, Marin JJ, Marzioni M, Bujanda L, LaRusso NF, et al. Polycystic liver diseases: advanced insights into the molecular mechanisms. Nat Rev Gastroenterol Hepatol. 2014;11(12):750–61. 25266109 10.1038/nrgastro.2014.155 PMC4526263

[5] Ubara Y, Takei R, Hoshino J, Tagami T, Sawa N, Yokota M, et al. Intravascular embolization therapy in a patient with an enlarged polycystic liver. Am J Kidney Dis. 2004;43(4):733–8. 15042552 10.1053/j.ajkd.2003.12.035

[6] Mekeel KL, Moss AA, Reddy KS, Douglas DD, Vargas HE, Carey EJ, et al. Living donor liver transplantation in polycystic liver disease. Liver Transpl. 2008;14(5):680–3. 18433036 10.1002/lt.21423

[7] Schiano TD, Bodian C, Schwartz ME, Glajchen N, Min AD. Accuracy and significance of computed tomographic scan assessment of hepatic volume in patients undergoing liver transplantation. Transplantation. 2000;69(4):545–50. 10708109 10.1097/00007890-200002270-00014

[8] Suzuki K, Epstein ML, Kohlbrenner R, Garg S, Hori M, Oto A, et al. Quantitative radiology: automated CT liver volumetry compared with interactive volumetry and manual volumetry. AJR Am J Roentgenol. 2011;197(4):W706–12. 21940543 10.2214/AJR.10.5958 PMC4277944

[9] Seppelt D, Ittermann T, Kromrey ML, Kolb C, vWahsen C, Heiss P, et al. Simple diameter measurement as predictor of liver volume and liver parenchymal disease. Sci Rep. 2022;12(1):1257. 35075169 10.1038/s41598-022-04825-8 PMC8786943

[10] Roloff AM, Heiss P, Schneider TP, Quadrat A, Kromrey ML, Zeman F, et al. Accuracy of simple approaches to assessing liver volume in radiological imaging. Abdom Radiol (NY). 2016;41(7):1293–9. 26907711 10.1007/s00261-016-0672-4

[11] Simpson AL, Geller DA, Hemming AW, Jarnagin WR, Clements LW, D’Angelica MI, et al. Liver planning software accurately predicts postoperative liver volume and measures early regeneration. J Am Coll Surg. 2014;219(2):199–207. 24862883 10.1016/j.jamcollsurg.2014.02.027 PMC4128572

[12] Bland JM, Altman DG. Statistical methods for assessing agreement between two methods of clinical measurement. Lancet. 1986;1(8476):307–10. 2868172

[13] Wang Z, Zhang C, Jiao T, Gao M, Zou G. Fully automatic segmentation and three-dimensional reconstruction of the liver in CT images. J Healthc Eng. 2018;2018:6797102. 30581550 10.1155/2018/6797102 PMC6276449

[14] Huang Q, Ding H, Wang X, Wang G. Fully automatic liver segmentation in CT images using modified graph cuts and feature detection. Comput Biol Med. 2018;95:198–208. 29524804 10.1016/j.compbiomed.2018.02.012

[15] Gul S, Khan MS, Bibi A, Khandakar A, Ayari MA, Chowdhury MEH. Deep learning techniques for liver and liver tumor segmentation: a review. Comput Biol Med. 2022;147: 105620. 35667155 10.1016/j.compbiomed.2022.105620

[16] Jin R, Wang M, Xu L, Lu J, Song E, Ma G. Automatic 3D CT liver segmentation based on fast global minimization of probabilistic active contour. Med Phys. 2023;50(4):2100–20. 36413182 10.1002/mp.16116

[17] Zoli M, Pisi P, Marchesini G, Bianchi GP, Turci GA, Pisi E. A rapid method for the in vivo measurement of liver volume. Liver. 1989;9(3):159–63. 2664394 10.1111/j.1600-0676.1989.tb00393.x

[18] Kitajima K, Taboury J, Boleslawski E, Savier E, Vaillant JC, Hannoun L. Sonographic preoperative assessment of liver volume before major liver resection. Gastroenterol Clin Biol. 2008;32(4):382–9. 18403156 10.1016/j.gcb.2008.02.007

