# Supplementary figures and images for: Development and validation of a simplified CT volumetry for estimating total liver volume in patients with autosomal dominant polycystic kidney and liver disease
Source: Clin Exp Nephrol. 2025 Jul 23;29(12):1754–63. doi: 10.1007/s10157-025-02721-9 (PMC12660425; doi:10.1007/s10157-025-02721-9)

## Slide 1
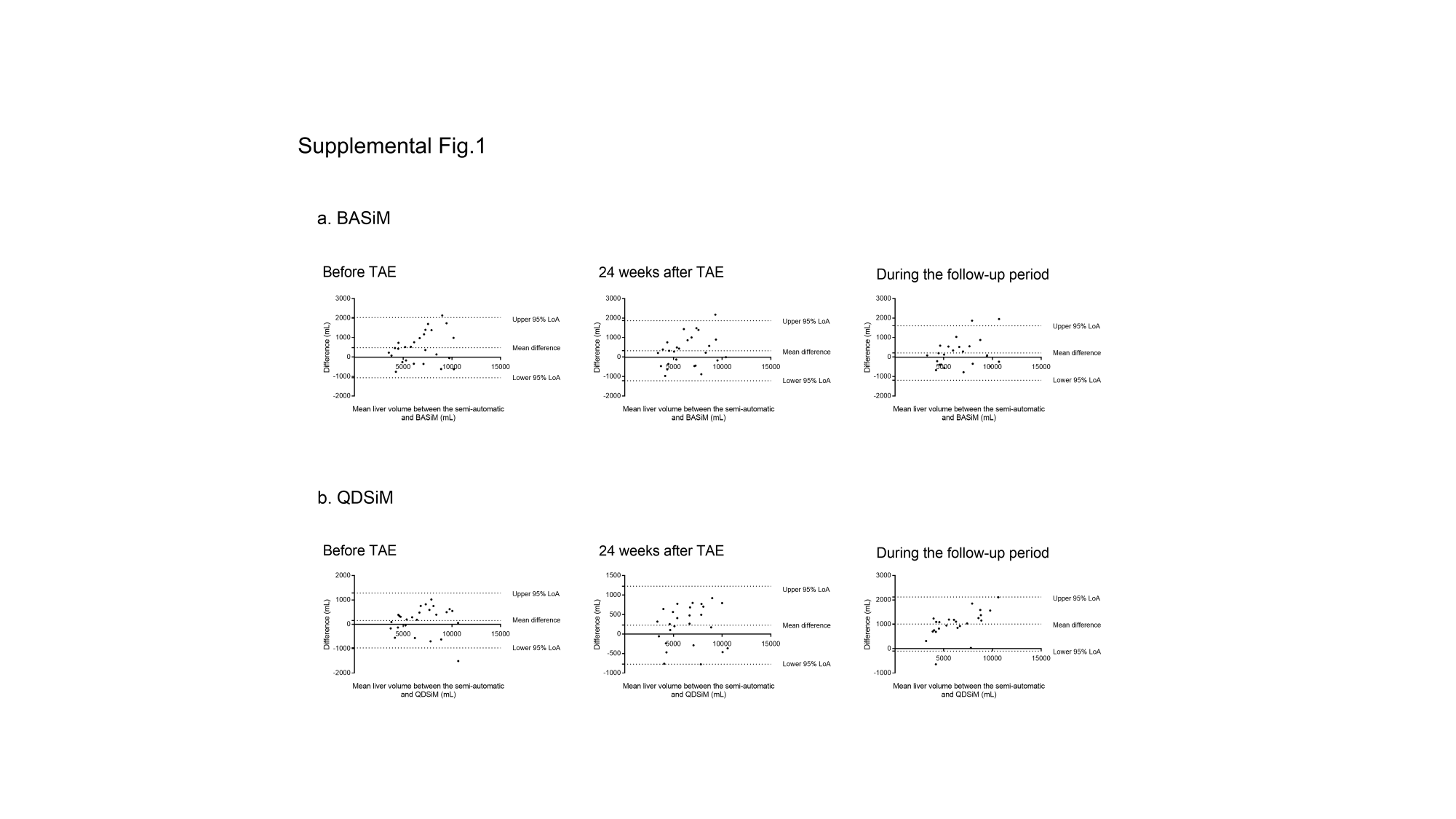

Supplement: Supplementary file 1 — Supplemental Fig. 1 Bland–Altman plots for agreement between liver volumes measured by a semi-automatic volumetry and two simple measurement methods: the Bi-axial Simplified Measurement Method (BASiM) (a) and the Quadri-Dimensional Simplified Measurement Method (QDSiM) (b) at three time points: before TAE, 24 weeks after TAE, and during the follow-up period. The mean difference in liver volume between a semi-automatic volumetry and BASiM (mean value between assessors A and B) was 482.3 mL before TAE, 324.1 mL 24 weeks after TAE and 209 mL during the follow-up period, respectively. The mean difference in liver volume between a semi-automatic volumetry and QDSiM (mean value between assessors A and B) was 156.0 mL before TAE, 228.3 mL 24 weeks after TAE and 1011 mL during the follow-up period, respectively. The 95% limits of agreement (LoA) are defined as the mean difference ± 1.96 standard deviations (SD) [file 10157_2025_2721_MOESM1_ESM.pptx]

## Slide 1
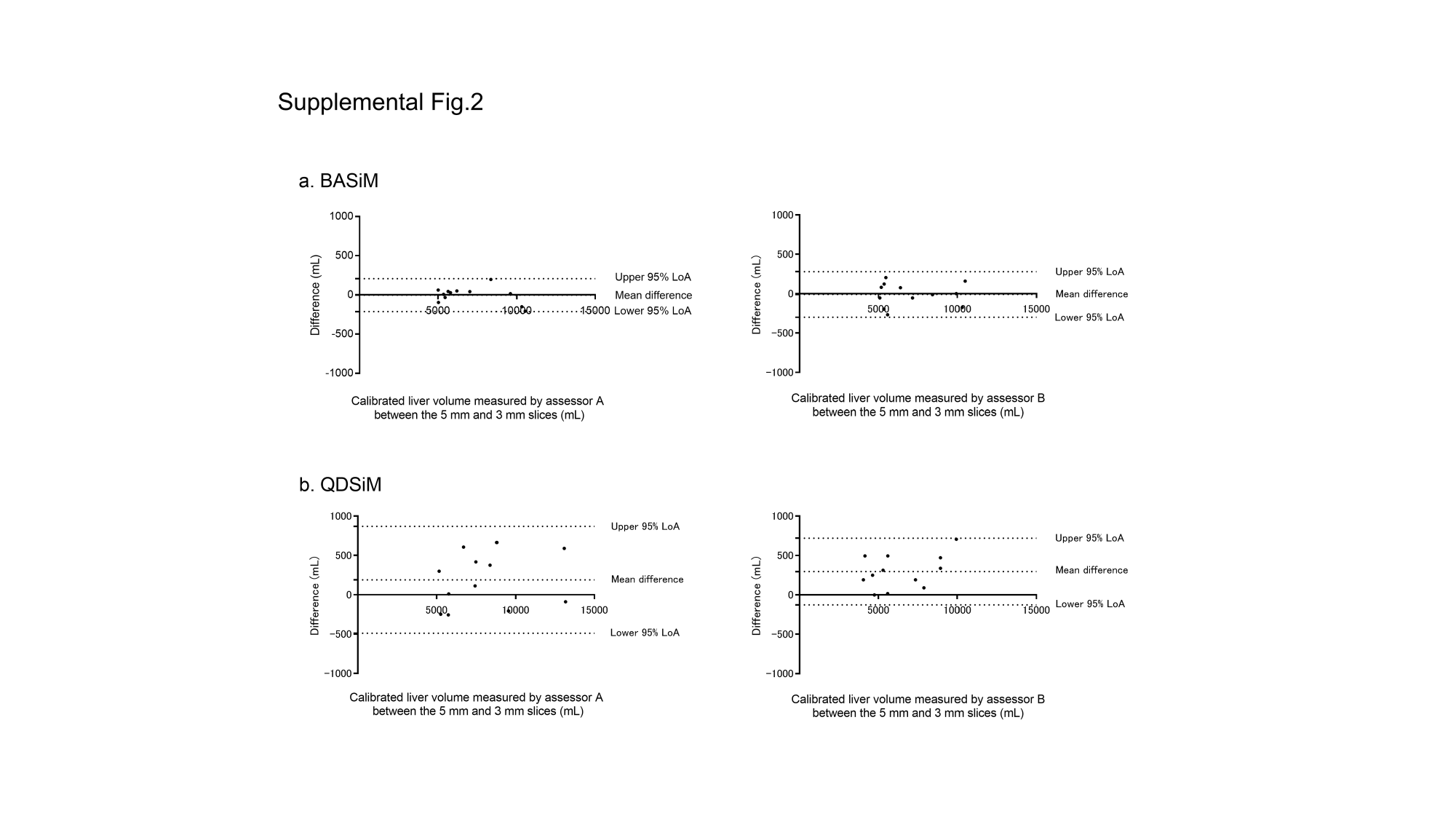

Supplement: Supplementary file 2 — Supplemental Fig. 2 Bland–Altman plots showing the agreement of calibrated liver volume measurements between 5 mm and 3 mm slices using BASiM (a) and QDSiM (b). In BASiM, the mean difference in calibrated liver volume between the 5 mm and 3 mm slices measured by Assessor A was – 2.84 mL, and by Assessor B was – 9.74 mL. In contrast, in QDSiM, the mean difference in calibrated liver volume between the 5 mm and 3 mm slices measured by Assessor A was 189.7 mL, and by Assessor B was 295.7 mL. The results show a high consistency in liver volume values calculated by BASiM for both 5 mm and 3 mm slices. In contrast, QDSiM demonstrates a lower consistency compared to BASiM. The 95% LoA are defined as the mean difference ± 1.96 SD [file 10157_2025_2721_MOESM2_ESM.pptx]
