# Supplementary material for: Development and validation of a simplified CT volumetry for estimating total liver volume in patients with autosomal dominant polycystic kidney and liver disease
Source: Clin Exp Nephrol. 2025 Jul 23;29(12):1754–63. doi: 10.1007/s10157-025-02721-9 (PMC12660425; doi:10.1007/s10157-025-02721-9)
Supplement: Supplementary file 3 — Supplementary file3 (DOCX 58 KB) [file 10157_2025_2721_MOESM3_ESM.docx]

**Supplemental Table 1. A detailed summary of patient characteristics, including CT slice thickness, observation periods, measurement methods and assessor measurement values.**

1. **BASiM and semi-automated volumetry**

| Observation periods | Patient's number | Slice thickness (cm) | Assessor A | | | | Assessor B | | | | Semi-automated volumetry |
| --- | --- | --- | --- | --- | --- | --- | --- | --- | --- | --- | --- |
|  |  |  | CC (cm) | AP (cm) | ML (cm) | LV (mL) | CC (cm) | AP (cm) | ML (cm) | LV (mL) | LV (mL) |
| Before TAE | No.1 | 5 | 26.7 | 22.1 | 27.8 | 5468.0 | 26.4 | 22.0 | 27.4 | 5304.6 | 5212.0 |
|  | No.2 | 5 | 40.5 | 24.7 | 31.9 | 10637.1 | 40.5 | 24.3 | 31.7 | 10399.2 | 9897.0 |
|  | No.3 | 5 | 27.9 | 20.2 | 26.5 | 4978.3 | 28.2 | 20.7 | 26.3 | 5117.5 | 4792.0 |
|  | No.4 | 5 | 30.3 | 21.7 | 28.2 | 6180.6 | 30.9 | 22.2 | 27.8 | 6356.7 | 5925.0 |
|  | No.5 | 5 | 27.9 | 19.7 | 30.7 | 5624.5 | 27.6 | 19.4 | 30.5 | 5443.6 | 6062.0 |
|  | No.6 | 5 | 24.3 | 24.7 | 28.9 | 5782.0 | 24.0 | 24.7 | 28.9 | 5710.6 | 6507.0 |
|  | No.7 | 5 | 34.8 | 21.8 | 32.7 | 8269.2 | 34.5 | 22.7 | 32.3 | 8431.9 | 8485.0 |
|  | No.8 | 5 | 33.6 | 25.7 | 33.3 | 9585.1 | 33.6 | 25.5 | 34.8 | 9938.9 | 9709.0 |
|  | No.9 | 5 | 39.3 | 19.6 | 27.3 | 7009.5 | 39.6 | 20.1 | 27.1 | 7190.2 | 7462.0 |
|  | No.10 | 3 | 21.6 | 19.7 | 27.1 | 3843.9 | 21.6 | 20.5 | 27.2 | 4014.7 | 4394.0 |
|  | No.11 | 5 | 28.5 | 22.5 | 30.5 | 6519.4 | 28.5 | 23.3 | 30.1 | 6662.6 | 7990.0 |
|  | No.12 | 5 | 37.0 | 23.3 | 29.8 | 8563.5 | 38.5 | 23.2 | 28.9 | 8604.5 | 10319.0 |
|  | No.13 | 5 | 32.5 | 22.8 | 27.3 | 6743.1 | 33.0 | 22.8 | 26.6 | 6671.3 | 8406.0 |
|  | No.14 | 5 | 29.0 | 21.6 | 29.7 | 6201.4 | 29.0 | 21.7 | 29.6 | 6209.1 | 7180.0 |
|  | No.15 | 5 | 35.5 | 23.6 | 34.1 | 9523.0 | 36.0 | 24.3 | 33.7 | 9826.9 | 10662.0 |
|  | No.16 | 5 | 29.0 | 17.3 | 25.2 | 4214.3 | 29.5 | 17.1 | 24.4 | 4102.9 | 4890.0 |
|  | No.17 | 3 | 25.8 | 17.8 | 26.6 | 4071.9 | 25.8 | 19.4 | 26.9 | 4488.0 | 4707.0 |
|  | No.18 | 5 | 28.0 | 20.1 | 26.8 | 5027.7 | 28.0 | 19.7 | 26.5 | 4872.5 | 5464.0 |
|  | No.19 | 5 | 38.0 | 19.8 | 26.2 | 6571.0 | 39.0 | 19.7 | 25.6 | 6556.2 | 7734.0 |
|  | No.20 | 5 | 35.0 | 22.2 | 28.4 | 7355.6 | 35.0 | 22.0 | 27.6 | 7084.0 | 8602.0 |
|  | No.21 | 5 | 23.5 | 17.5 | 24.9 | 3413.4 | 23.5 | 17.7 | 24.6 | 3410.8 | 3640.0 |
|  | No.22 | 5 | 24.9 | 20.6 | 26.7 | 4565.2 | 25.0 | 20.8 | 27.1 | 4697.3 | 3874.0 |
|  | No.23 | 5 | 32.0 | 22.9 | 30.2 | 7376.9 | 31.0 | 23.0 | 30.1 | 7153.8 | 6915.0 |
|  | No.24 | 3 | 25.8 | 18.2 | 24.7 | 3866.0 | 25.5 | 17.6 | 24.5 | 3665.2 | 3843.0 |
|  | No.25 | 3 | 36.0 | 23.2 | 33.7 | 9382.1 | 35.7 | 23.0 | 33.0 | 9032.1 | 8592.0 |
|  | No.26 | 3 | 33.9 | 23.2 | 30.6 | 8022.1 | 33.3 | 23.4 | 30.3 | 7870.1 | 10083.0 |
| 24 weeks after TAE | No.1 | 5 | 27.5 | 21.3 | 27.4 | 5349.9 | 27.0 | 22.1 | 27.0 | 5370.3 | 5233.0 |
|  | No.2 | 5 | 41.7 | 24.2 | 30.9 | 10394.1 | 42.0 | 24.0 | 30.9 | 10382.4 | 10382.0 |
|  | No.3 | 5 | 26.0 | 19.9 | 26.7 | 4604.9 | 26.5 | 20.4 | 26.1 | 4703.2 | 4296.0 |
|  | No.4 | 5 | 26.4 | 19.2 | 27.2 | 4595.7 | 26.1 | 20.2 | 27.2 | 4780.1 | 4057.0 |
|  | No.5 | 3 | 28.5 | 18.6 | 30.6 | 5407.0 | 27.9 | 19.1 | 29.9 | 5311.1 | 5796.0 |
|  | No.6 | 5 | 22.5 | 23.5 | 28.8 | 5076.0 | 22.5 | 24.2 | 28.3 | 5136.5 | 5598.0 |
|  | No.7 | 3 | 34.5 | 22.0 | 32.4 | 8197.2 | 34.5 | 22.3 | 32.0 | 8206.4 | 8423.0 |
|  | No.8 | 3 | 33.9 | 25.3 | 33.1 | 9463.0 | 33.9 | 25.7 | 33.6 | 9757.8 | 9437.0 |
|  | No.9 | 3 | 39.3 | 20.1 | 28.0 | 7372.7 | 39.3 | 20.6 | 27.4 | 7394.2 | 6922.0 |
|  | No.10 | 3 | 21.8 | 18.9 | 27.1 | 3721.9 | 21.9 | 19.3 | 26.4 | 3719.5 | 4109.0 |
|  | No.11 | 5 | 28.5 | 22.4 | 30.7 | 6533.0 | 28.5 | 23.3 | 30.3 | 6706.9 | 8103.0 |
|  | No.12 | 5 | 37.5 | 22.1 | 29.9 | 8259.9 | 38.0 | 22.4 | 30.0 | 8512.0 | 8957.0 |
|  | No.13 | 5 | 33.5 | 22.7 | 26.8 | 6793.4 | 34.0 | 22.8 | 26.9 | 6951.0 | 8267.0 |
|  | No.14 | 5 | 28.5 | 21.5 | 29.1 | 5943.7 | 28.5 | 22.4 | 28.7 | 6107.4 | 6891.0 |
|  | No.15 | 5 | 33.5 | 22.9 | 34.0 | 8694.4 | 34.5 | 23.5 | 33.8 | 9134.5 | 9813.0 |
|  | No.16 | 5 | 29.0 | 16.7 | 25.0 | 4035.8 | 29.0 | 16.5 | 24.7 | 3939.7 | 4746.0 |
|  | No.17 | 3 | 26.0 | 17.5 | 27.1 | 4110.2 | 25.7 | 20.1 | 27.2 | 4683.6 | 4719.0 |
|  | No.18 | 5 | 28.0 | 20.2 | 26.2 | 4939.6 | 28.0 | 20.0 | 26.3 | 4909.3 | 5205.0 |
|  | No.19 | 5 | 36.5 | 20.2 | 26.0 | 6389.9 | 35.0 | 20.8 | 26.1 | 6333.6 | 7368.0 |
|  | No.20 | 5 | 28.0 | 20.9 | 27.2 | 5305.8 | 28.0 | 21.3 | 27.2 | 5407.4 | 6790.0 |
|  | No.21 | 5 | 23.0 | 17.3 | 24.6 | 3262.8 | 23.5 | 17.6 | 24.3 | 3350.2 | 3518.0 |
|  | No.22 | 3 | 24.9 | 21.0 | 26.2 | 4566.7 | 24.3 | 21.4 | 27.2 | 4714.8 | 3671.0 |
|  | No.23 | 3 | 32.7 | 22.7 | 30.4 | 7521.9 | 31.2 | 23.5 | 30.5 | 7454.2 | 7049.0 |
|  | No.24 | 3 | 25.2 | 18.4 | 25.6 | 3956.7 | 25.2 | 18.7 | 25.1 | 3942.7 | 3486.0 |
|  | No.25 | 3 | 32.7 | 22.8 | 33.1 | 8226.0 | 32.7 | 23.1 | 33.3 | 8384.6 | 7424.0 |
|  | No.26 | 3 | 33.0 | 23.6 | 30.8 | 7995.7 | 33.6 | 24.7 | 30.5 | 8437.5 | 10392.0 |
| During the follow-up period | No.1 | 5 | 27.0 | 21.7 | 27.1 | 5292.6 | 27.0 | 21.9 | 27.3 | 5380.8 | 4776.5 |
|  | No.2 | 5 | 40.0 | 25.0 | 33.0 | 11000.0 | 40.0 | 24.2 | 32.8 | 10583.5 | 10551.6 |
|  | No.3 | 5 | 27.5 | 19.6 | 26.7 | 4797.1 | 27.0 | 20.0 | 26.5 | 4770.0 | 4390.2 |
|  | No.4 | 5 | 22.5 | 20.9 | 27.2 | 4263.6 | 24.5 | 21.0 | 26.8 | 4596.2 | 4220.1 |
|  | No.5 | 5 | 25.0 | 19.3 | 30.8 | 4953.7 | 25.0 | 19.4 | 30.3 | 4898.5 | 4538.9 |
|  | No.6 | 5 | 23.5 | 22.9 | 29.2 | 5238.0 | 23.5 | 22.8 | 29.1 | 5197.3 | 5758.4 |
|  | No.7 | 5 | 31.5 | 21.7 | 31.0 | 7063.4 | 31.5 | 21.0 | 30.0 | 6615.0 | 7124.7 |
|  | No.8 | 3 | 34.2 | 25.9 | 34.5 | 10186.5 | 34.2 | 25.7 | 33.7 | 9873.4 | 9551.8 |
|  | No.9 | 5 | 41.0 | 21.4 | 28.0 | 8189.1 | 40.5 | 21.6 | 27.8 | 8106.5 | 7803.4 |
|  | No.10 | 5 | 27.5 | 18.8 | 27.4 | 4721.9 | 22.5 | 19.6 | 27.5 | 4042.5 | 4580.0 |
|  | No.11 | 5 | 28.0 | 24.3 | 33.2 | 7529.8 | 28.0 | 24.5 | 31.5 | 7203.0 | 7918.7 |
|  | No.12 | 3 | 37.0 | 25.1 | 30.0 | 9287.0 | 36.5 | 25.6 | 30.6 | 9530.9 | 9500.0 |
|  | No.13 | 5 | 32.0 | 24.1 | 27.3 | 7017.9 | 32.5 | 24.0 | 26.7 | 6942.0 | 8848.0 |
|  | No.14 | 5 | 30.5 | 21.6 | 28.5 | 6258.6 | 31.0 | 22.0 | 28.2 | 6410.8 | 6852.3 |
|  | No.15 | 5 | 32.0 | 24.9 | 33.7 | 8950.7 | 34.0 | 26.2 | 33.3 | 9887.9 | 9436.6 |
|  | No.16 | 5 | 28.5 | 17.2 | 26.5 | 4330.1 | 29.0 | 17.4 | 25.9 | 4356.4 | 4929.3 |
|  | No.17 | 5 | 27.0 | 18.8 | 27.1 | 4585.3 | 27.3 | 20.7 | 28.5 | 5368.5 | 5102.1 |
|  | No.18 | 5 | 29.5 | 21.8 | 27.9 | 5980.8 | 29.0 | 21.3 | 27.2 | 5600.5 | 6130.8 |
|  | No.19 | 5 | 37.0 | 23.4 | 28.5 | 8225.1 | 37.5 | 23.6 | 28.5 | 8407.5 | 9194.5 |
|  | No.20 | 5 | 27.5 | 22.6 | 28.2 | 5842.1 | 27.0 | 22.6 | 28.1 | 5715.5 | 6815.4 |
|  | No.21 | 5 | 23.0 | 17.6 | 24.1 | 3251.9 | 22.5 | 18.2 | 24.0 | 3276.0 | 3350.5 |
|  | No.22 | 5 | 24.5 | 21.0 | 27.2 | 4664.8 | 24.0 | 20.5 | 27.0 | 4428.0 | 3877.5 |
|  | No.23 | 5 | 31.0 | 23.1 | 30.2 | 7208.7 | 32.0 | 23.9 | 30.0 | 7648.0 | 6651.1 |
|  | No.24 |  |  |  |  |  |  |  |  |  |  |
|  | No.25 |  |  |  |  |  |  |  |  |  |  |
|  | No.26 | 5 | 34.2 | 25.9 | 32.0 | 9448.3 | 34.0 | 28.0 | 31.4 | 9964.3 | 11657.7 |

1. **QDSiM**

| Observation periods | Patient's number | Slice thickness (cm) | Assessor A | | | | | | Assessor B | | | | | |
| --- | --- | --- | --- | --- | --- | --- | --- | --- | --- | --- | --- | --- | --- | --- |
|  |  |  | RCC (cm) | RAP (cm) | LCC (cm) | LAP (mL) | ML (mL) | LV  (mL) | RCC (cm) | RAP (cm) | LCC (cm) | LAP (mL) | ML (mL) | LV (mL) |
| Before TAE | No.1 | 5 | 26.5 | 20.8 | 15.0 | 19.7 | 27.3 | 5768.1 | 26.4 | 20.4 | 11.8 | 14.4 | 26.8 | 4746.8 |
|  | No.2 | 5 | 40.5 | 21.9 | 35.5 | 22.0 | 31.7 | 13216.8 | 40.5 | 19.7 | 13.7 | 34.5 | 30.2 | 9592.3 |
|  | No.3 | 5 | 27.9 | 18.2 | 15.6 | 18.5 | 25.2 | 5009.0 | 26.7 | 17.8 | 11.3 | 15 | 24.2 | 3900.8 |
|  | No.4 | 5 | 31.0 | 18.6 | 24.5 | 20.1 | 27.7 | 7392.2 | 30.0 | 18.3 | 11.7 | 24.3 | 26.8 | 5583.2 |
|  | No.5 | 5 | 28.0 | 18.7 | 18.5 | 18.2 | 29.8 | 6410.7 | 26.7 | 18.3 | 10.9 | 19.5 | 29.3 | 5136 |
|  | No.6 | 5 | 24.5 | 22.4 | 20.0 | 22.5 | 29.2 | 7283.5 | 21.3 | 20.6 | 14.3 | 20.4 | 29.3 | 5350.9 |
|  | No.7 | 5 | 34.5 | 19.6 | 18.5 | 19.8 | 31.5 | 8207.9 | 34.2 | 20.1 | 13.4 | 17.7 | 31.4 | 7258.1 |
|  | No.8 | 5 | 33.6 | 24.3 | 17.5 | 22.8 | 31.8 | 9649.7 | 33.0 | 24.5 | 21.8 | 17.1 | 29.7 | 8771 |
|  | No.9 | 5 | 38.5 | 17.6 | 29.0 | 18.7 | 27.8 | 8478.0 | 39.0 | 18.1 | 14.5 | 30 | 27.5 | 7843.7 |
|  | No.10 | 3 | 21.0 | 17.6 | 19.5 | 17.9 | 27.9 | 5011.0 | 20.7 | 17.7 | 12.3 | 18.9 | 27 | 4042.3 |
|  | No.11 | 5 | 28.5 | 20.2 | 22.5 | 21.3 | 30.6 | 8061.4 | 27.5 | 20.4 | 14.9 | 22 | 30.3 | 6732.7 |
|  | No.12 | 5 | 38.5 | 19.7 | 29.5 | 19.3 | 29.7 | 9874.5 | 37.5 | 19.3 | 17.8 | 32 | 29.9 | 9667.8 |
|  | No.13 | 5 | 31.0 | 20.4 | 30.0 | 19.0 | 27.3 | 8193.2 | 31.5 | 18.8 | 13.2 | 29 | 27 | 6581.3 |
|  | No.14 | 5 | 29.0 | 17.9 | 22.5 | 17.7 | 30.1 | 6892.3 | 28.0 | 19.1 | 12.5 | 22.5 | 29.2 | 5957.2 |
|  | No.15 | 5 | 34.0 | 19.7 | 33.0 | 22.6 | 33.0 | 11662.1 | 33.0 | 18.0 | 18.3 | 31 | 32.9 | 9551.7 |
|  | No.16 | 5 | 28.5 | 15.8 | 20.5 | 15.1 | 25.7 | 4890.2 | 28.5 | 15.5 | 11.4 | 21 | 25.1 | 4274.2 |
|  | No.17 | 3 | 25.2 | 19.0 | 13.0 | 16.8 | 26.3 | 4589.5 | 24.9 | 17.2 | 13.3 | 13.2 | 26.8 | 4045.7 |
|  | No.18 | 5 | 27.0 | 17.6 | 24.0 | 17.1 | 25.7 | 5696.8 | 27.0 | 17.4 | 12.3 | 23 | 25.7 | 4836.1 |
|  | No.19 | 5 | 37.0 | 17.6 | 26.5 | 17.1 | 26.5 | 7321.3 | 37.0 | 18.2 | 11.5 | 26.5 | 26.6 | 6504.7 |
|  | No.20 | 5 | 35.0 | 20.2 | 25.5 | 19.2 | 28.3 | 8468.9 | 34.0 | 21.0 | 17 | 25 | 27.9 | 7944.5 |
|  | No.21 | 5 | 23.5 | 15.6 | 18.5 | 15.6 | 24.9 | 4077.5 | 22.5 | 15.6 | 12.7 | 17.5 | 24.7 | 3539.8 |
|  | No.22 | 5 | 25.5 | 19.0 | 11.0 | 19.2 | 26.6 | 4626.6 | 24.0 | 18.9 | 18.1 | 11 | 25.9 | 4226.2 |
|  | No.23 | 5 | 30.5 | 20.6 | 21.9 | 21.2 | 25.7 | 7008.1 | 31.0 | 21.0 | 13.8 | 23 | 24.2 | 5858.8 |
|  | No.24 | 3 | 25.5 | 17.7 | 14.1 | 16.2 | 24.1 | 4100.7 | 25.5 | 17.4 | 10.4 | 14.1 | 23 | 3394.5 |
|  | No.25 | 3 | 35.4 | 20.4 | 26.7 | 20.4 | 32.8 | 10374.5 | 34.8 | 18.1 | 14.3 | 27.9 | 31.3 | 8050.8 |
|  | No.26 | 3 | 32.4 | 21.0 | 27.6 | 21.0 | 30.6 | 9641.5 | 31.8 | 21.9 | 19 | 27 | 30.7 | 9282.3 |
| 24 weeks after TAE | No.1 | 5 | 27.0 | 19.5 | 15.6 | 18.9 | 26.2 | 5388.0 | 24.7 | 20.0 | 10.2 | 14 | 24.8 | 3948.2 |
|  | No.2 | 5 | 41.5 | 22.1 | 35.0 | 21.9 | 30.4 | 12791.1 | 39.5 | 18.4 | 13.7 | 34 | 29.2 | 8706 |
|  | No.3 | 5 | 26.6 | 16.5 | 9.5 | 17.4 | 25.2 | 3800.4 | 26.0 | 18.6 | 10.9 | 9 | 24.1 | 3504.7 |
|  | No.4 | 5 | 24.3 | 16.9 | 24.0 | 17.6 | 25.8 | 5379.3 | 23.7 | 15.8 | 9.3 | 23.7 | 24.7 | 3673.3 |
|  | No.5 | 3 | 28.2 | 18.2 | 16.2 | 18.0 | 28.9 | 5813.0 | 22.2 | 18.1 | 10.3 | 19.2 | 28.2 | 4227 |
|  | No.6 | 5 | 22.5 | 20.6 | 18.0 | 21.6 | 27.6 | 5871.7 | 22.0 | 19.5 | 12.5 | 17 | 28.1 | 4506.5 |
|  | No.7 | 3 | 34.2 | 19.7 | 19.2 | 19.3 | 31.4 | 8188.4 | 33.9 | 20.1 | 13.2 | 18.6 | 31.3 | 7253.1 |
|  | No.8 | 3 | 33.9 | 23.0 | 16.5 | 22.7 | 31.3 | 9039.8 | 33.3 | 24.9 | 17 | 15.6 | 29.2 | 7988.9 |
|  | No.9 | 3 | 39.3 | 17.8 | 28.8 | 17.9 | 24.5 | 7449.2 | 38.7 | 18.5 | 12.8 | 28.2 | 25.9 | 6973 |
|  | No.10 | 3 | 21.0 | 17.5 | 19.5 | 17.5 | 26.9 | 4772.4 | 20.4 | 17.2 | 12.4 | 18.9 | 26.8 | 3921.1 |
|  | No.11 | 5 | 28.5 | 20.8 | 23.5 | 21.7 | 30.6 | 8422.8 | 27.5 | 20.5 | 15 | 22 | 30.4 | 6792.5 |
|  | No.12 | 5 | 37.5 | 16.4 | 29.5 | 20.1 | 29.8 | 9003.5 | 36.5 | 17.0 | 16.7 | 31 | 30.1 | 8565 |
|  | No.13 | 5 | 32.0 | 20.0 | 30.0 | 19.5 | 27.0 | 8259.3 | 32.5 | 18.7 | 13.2 | 29.5 | 27 | 6730.8 |
|  | No.14 | 5 | 28.5 | 18.7 | 22.5 | 18.6 | 29.6 | 7052.1 | 27.0 | 19.9 | 11.8 | 21.5 | 29.2 | 5774.3 |
|  | No.15 | 5 | 33.5 | 20.7 | 32.0 | 22.8 | 33.1 | 11759.4 | 32.5 | 17.7 | 16.2 | 30.5 | 32.9 | 8795.4 |
|  | No.16 | 5 | 29.0 | 15.6 | 20.5 | 15.0 | 25.1 | 4762.9 | 28.5 | 14.9 | 11.6 | 20 | 25.7 | 4219 |
|  | No.17 | 3 | 26.0 | 19.3 | 14.0 | 17.6 | 26.5 | 4951.2 | 24.5 | 19.1 | 12.6 | 13 | 27.1 | 4280.1 |
|  | No.18 | 5 | 27.8 | 17.4 | 22.0 | 17.7 | 25.2 | 5494.8 | 27.0 | 17.6 | 11.5 | 21 | 25.2 | 4515.2 |
|  | No.19 | 5 | 35.0 | 17.9 | 26.0 | 17.6 | 26.6 | 7200.1 | 34.5 | 17.8 | 11.2 | 25.5 | 26.4 | 5938 |
|  | No.20 | 5 | 28.0 | 18.9 | 25.5 | 18.1 | 27.5 | 6812.6 | 27.0 | 18.2 | 17.1 | 24.5 | 27.4 | 6235.9 |
|  | No.21 | 5 | 23.0 | 15.4 | 14.0 | 16.0 | 24.5 | 3538.6 | 22.5 | 15.4 | 11.6 | 10.2 | 24.6 | 2858.6 |
|  | No.22 | 3 | 24.6 | 20.0 | 11.4 | 19.5 | 26.5 | 4735.8 | 24.0 | 19.7 | 15.5 | 10.5 | 26 | 4131.1 |
|  | No.23 | 3 | 31.8 | 20.2 | 18.6 | 20.9 | 25.6 | 6605.0 | 31.5 | 21.0 | 13.6 | 23.7 | 24.9 | 6124.3 |
|  | No.24 | 3 | 24.9 | 17.6 | 13.8 | 17.3 | 23.9 | 4045.0 | 24.6 | 17.5 | 10.1 | 12.9 | 21.7 | 3042.3 |
|  | No.25 | 3 | 32.7 | 20.0 | 25.2 | 19.7 | 31.8 | 9131.9 | 32.1 | 18.0 | 14.3 | 25.8 | 30.7 | 7266.2 |
|  | No.26 | 3 | 33.3 | 21.1 | 28.2 | 20.8 | 30.8 | 9938.0 | 32.4 | 22.0 | 18.3 | 27.6 | 30.4 | 9255.9 |
| During the follow-up period | No.1 | 5 | 27.0 | 19.2 | 9.5 | 8.7 | 21.7 | 3261.8 | 24.5 | 19.6 | 13.2 | 11.9 | 25.7 | 4094.5 |
|  | No.2 | 5 | 40.0 | 19.6 | 26.0 | 15.7 | 32.2 | 9611.2 | 38.0 | 20.0 | 16.2 | 17.6 | 32 | 8361 |
|  | No.3 | 5 | 28.0 | 17.2 | 11.0 | 11.8 | 25.3 | 3866.6 | 22.5 | 18.0 | 13 | 11.4 | 24.6 | 3402.2 |
|  | No.4 | 5 | 22.5 | 17.1 | 22.0 | 11.2 | 25.4 | 4012.8 | 19.1 | 17.6 | 12 | 12.1 | 25.1 | 3020.5 |
|  | No.5 | 5 | 25.0 | 18.5 | 15.0 | 10.4 | 26.4 | 4094.3 | 21.2 | 18.4 | 11.2 | 15.6 | 25.5 | 3600.6 |
|  | No.6 | 5 | 23.5 | 19.4 | 18.5 | 13.4 | 29.1 | 5115.2 | 20.5 | 19.8 | 14 | 15.6 | 28.8 | 4495 |
|  | No.7 | 5 | 31.0 | 18.7 | 22.0 | 12.4 | 30.6 | 6537.1 | 28.9 | 18.2 | 12.6 | 19 | 30.6 | 5855.2 |
|  | No.8 | 3 | 33.9 | 23.9 | 16.2 | 13.1 | 31.6 | 8075.1 | 32.0 | 25.0 | 15.1 | 15.2 | 30.5 | 7850.1 |
|  | No.9 | 5 | 41.0 | 18.7 | 30.5 | 14.0 | 27.1 | 8079.8 | 37.9 | 19.4 | 14.4 | 23.8 | 27.7 | 7465 |
|  | No.10 | 5 | 22.5 | 17.8 | 20.5 | 1.2 | 27.0 | 2858.9 | 20.2 | 17.7 | 12.3 | 17.3 | 26.8 | 3821.2 |
|  | No.11 | 5 | 27.5 | 21.5 | 22.0 | 13.2 | 32.4 | 7140.4 | 24.4 | 21.2 | 16 | 18.8 | 32.4 | 6626.4 |
|  | No.12 | 3 | 37.0 | 18.4 | 24.5 | 16.1 | 30.5 | 8197.4 | 34.7 | 18.9 | 18.3 | 25.5 | 28.7 | 8053.8 |
|  | No.13 | 5 | 32.0 | 20.1 | 29.5 | 14.4 | 27.4 | 7318.9 | 30.0 | 20.8 | 14 | 27.4 | 26.5 | 6675.4 |
|  | No.14 | 5 | 29.5 | 19.0 | 23.0 | 13.0 | 28.6 | 6145.9 | 27.4 | 19.4 | 15.1 | 20.7 | 27.7 | 5845.6 |
|  | No.15 | 5 | 32.5 | 16.8 | 31.5 | 17.0 | 33.8 | 9156.4 | 30.8 | 15.4 | 15.7 | 26.3 | 33.4 | 7408.4 |
|  | No.16 | 5 | 29.0 | 15.3 | 21.0 | 9.1 | 25.8 | 4084.5 | 27.7 | 15.1 | 10.5 | 21.5 | 25.6 | 4121.7 |
|  | No.17 | 5 | 27.0 | 19.7 | 10.5 | 11.1 | 25.3 | 4111.5 | 22.6 | 16.9 | 14.9 | 13.9 | 26.6 | 3917.2 |
|  | No.18 | 5 | 29.0 | 17.8 | 23.5 | 12.6 | 25.4 | 5163.1 | 26.4 | 17.5 | 12.7 | 21.8 | 25.5 | 4710.2 |
|  | No.19 | 5 | 40.0 | 20.5 | 27.0 | 12.6 | 28.4 | 8235.2 | 37.7 | 20.0 | 14.2 | 23 | 28.3 | 7645.2 |
|  | No.20 | 5 | 26.5 | 17.4 | 27.0 | 11.5 | 28.5 | 5492.8 | 25.7 | 18.1 | 17 | 23.5 | 27.4 | 5923 |
|  | No.21 | 5 | 23.5 | 14.8 | 18.5 | 10.3 | 24.1 | 3242.1 | 19.6 | 15.3 | 10.7 | 15.9 | 24.1 | 2831.8 |
|  | No.22 | 5 | 26.5 | 19.4 | 12.9 | 17.8 | 27.0 | 5025.4 | 21.9 | 19.2 | 16.8 | 10.2 | 27.1 | 4009.7 |
|  | No.23 | 5 | 32.0 | 19.5 | 17.5 | 13.9 | 26.8 | 5816.6 | 27.2 | 19.0 | 12.3 | 24.5 | 25 | 5113.4 |
|  | No.24 |  |  |  |  |  |  |  |  |  |  |  |  |  |
|  | No.25 |  |  |  |  |  |  |  |  |  |  |  |  |  |
|  | No.26 | 5 | 35.5 | 23.0 | 29.5 | 17.4 | 31.6 | 10515.7 | 30.7 | 23.2 | 17.5 | 22.2 | 31.2 | 8585.8 |

**Supplemental Table 2. A detailed summary of patient characteristics (Data from 3 mm CT slices, limited to patients with both 5 mm and 3 mm slices)**

**A. BASiM**

| Observation periods | Patient's number | Slice thickness (cm) | Assessor A | | | | Assessor B | | | |
| --- | --- | --- | --- | --- | --- | --- | --- | --- | --- | --- |
|  |  |  | CC (cm) | AP (cm) | ML (cm) | LV (mL) | CC (cm) | AP (cm) | ML (cm) | LV (mL) |
| Before TAE | No.1 | 3 | 26.7 | 21.9 | 27.9 | 5435.4 | 26.7 | 22.5 | 27.1 | 5426.8 |
|  | No.2 | 3 | 40.5 | 24.2 | 31.9 | 10430.3 | 41.2 | 24.8 | 31.0 | 10558.2 |
|  | No.3 | 3 | 28.5 | 20.0 | 26.6 | 5040.5 | 27.9 | 20.7 | 26.3 | 5063.0 |
|  | No.4 | 3 | 31.2 | 21.1 | 28.3 | 6231.8 | 29.7 | 22.8 | 28.5 | 6433.0 |
|  | No.5 | 3 | 28.8 | 19.0 | 31.1 | 5669.4 | 27.3 | 18.6 | 31.0 | 5247.1 |
|  | No.6 | 3 | 24.6 | 24.4 | 29.0 | 5811.5 | 22.8 | 24.7 | 29.0 | 5443.9 |
|  | No.7 | 3 | 34.8 | 22.2 | 32.8 | 8467.4 | 34.8 | 22.4 | 32.4 | 8418.8 |
|  | No.8 | 3 | 33.6 | 25.3 | 33.9 | 9601.6 | 33.6 | 25.5 | 34.8 | 9938.9 |
|  | No.9 | 3 | 39.6 | 19.3 | 27.7 | 7051.8 | 39.3 | 20.1 | 27.1 | 7135.7 |
| 24 weeks after TAE | No.1 | 3 | 27.0 | 21.3 | 27.4 | 5349.9 | 27.0 | 22.1 | 27.0 | 5370.3 |
|  | No.2 | 3 | 42.0 | 24.2 | 30.9 | 10394.1 | 42.0 | 24.0 | 30.9 | 10382.4 |
|  | No.6 | 3 | 22.0 | 23.5 | 28.8 | 5076.0 | 22.5 | 24.2 | 28.3 | 5136.5 |

**Ｂ. QDSiM**

| Observation periods | Patient's number | Slice thickness (cm) | Assessor A | | | | | | Assessor B | | | | | |
| --- | --- | --- | --- | --- | --- | --- | --- | --- | --- | --- | --- | --- | --- | --- |
|  |  |  | RCC (cm) | RAP (cm) | LCC (cm) | LAP (mL) | ML (mL) | LV (mL) | RCC (cm) | RAP (cm) | LCC (cm) | LAP (mL) | ML (mL) | LV (mL) |
| Before TAE | No.1 | 5 | 26.7 | 20.0 | 15.0 | 19.6 | 28.0 | 5778.2 | 26.7 | 19.8 | 14.3 | 12.3 | 26.9 | 4745.3 |
|  | No.2 | 5 | 40.5 | 21.8 | 35.1 | 21.8 | 31.8 | 13128.0 | 39.8 | 20.2 | 33.9 | 15.4 | 31.0 | 10297.5 |
|  | No.3 | 5 | 29.4 | 18.3 | 15.9 | 18.5 | 25.5 | 5308.6 | 28.4 | 17.9 | 15.3 | 12.8 | 25.0 | 4395.7 |
|  | No.4 | 5 | 31.2 | 18.7 | 24.6 | 19.4 | 28.3 | 7504.3 | 30.6 | 18.4 | 24.3 | 11.8 | 26.4 | 5598.8 |
|  | No.5 | 5 | 28.8 | 18.9 | 20.1 | 18.0 | 31.0 | 7016.1 | 27.0 | 18.6 | 20.1 | 10.7 | 30.4 | 5449.1 |
|  | No.6 | 5 | 24.6 | 21.7 | 21.0 | 22.5 | 30.6 | 7701.8 | 23.4 | 20.5 | 20.7 | 15.1 | 29.6 | 5845.2 |
|  | No.7 | 5 | 34.8 | 20.2 | 18.6 | 19.4 | 32.3 | 8583.1 | 34.2 | 20.5 | 17.4 | 13.9 | 31.6 | 7448.1 |
|  | No.8 | 5 | 33.6 | 23.2 | 17.7 | 23.6 | 31.6 | 9443.5 | 33.3 | 24.3 | 17.4 | 21.8 | 30.7 | 9108.3 |
|  | No.9 | 5 | 39.6 | 18.3 | 30.9 | 19.2 | 27.7 | 9143.0 | 39.0 | 17.9 | 30.6 | 15.1 | 27.4 | 7933.0 |
| 24 weeks after TAE | No.1 | 5 | 27.0 | 19.6 | 14.7 | 18.3 | 25.7 | 5139.9 | 26.4 | 19.6 | 14.7 | 10.7 | 24.6 | 4139.5 |
|  | No.2 | 5 | 42.0 | 21.9 | 35.7 | 22.1 | 31.3 | 13380.8 | 40.1 | 19.0 | 34.3 | 14.1 | 29.4 | 9176.1 |
|  | No.6 | 5 | 21.0 | 20.6 | 17.7 | 21.6 | 27.6 | 5615.7 | 22.5 | 20.0 | 17.4 | 12.4 | 28.5 | 4755.2 |
